# Supplementary material for: Investigation of the Bimodal Leaching Response of RAM Chip Gold Fingers in Ammonia Thiosulfate Solution
Source: Materials (Basel). 2023 Jul 11;16(14):4940. doi: 10.3390/ma16144940 (PMC10381887; doi:10.3390/ma16144940)
Supplement: Supplementary file 1 [file materials-16-04940-s001.zip › materials-2455618-supplementary.pdf]

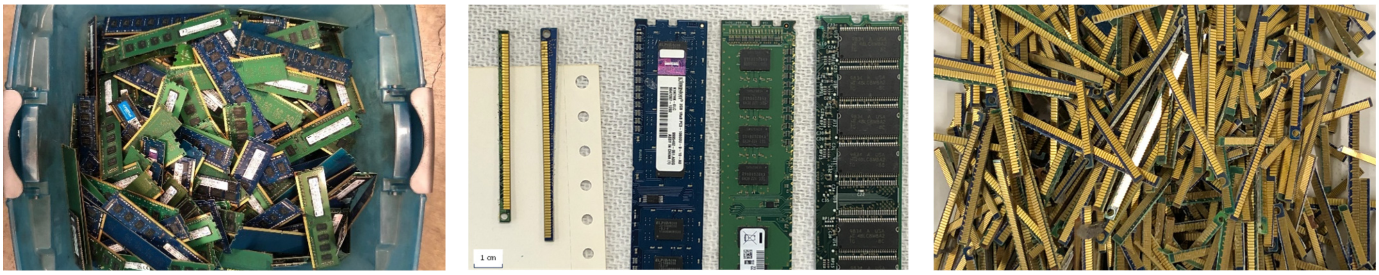

(a) **(b)** **(c)**

Figure S1. (a) As-received RAM chips, **(b)** stamped Au-fingers and remaining boards, **(c)** as-stamped untreated Au-fingers.

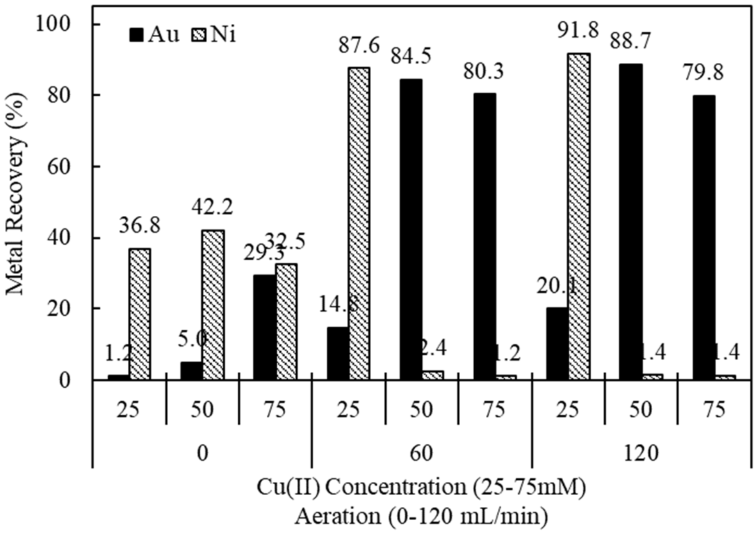

Figure S2. Recovery of Au and Ni at copper concentrations of 25 mM, 50mM, and 75 mM at the aeration rates of 0 mL/min, 60 mL/min, and 120 mL/min at 240 min (summary of Experiment 2–10).

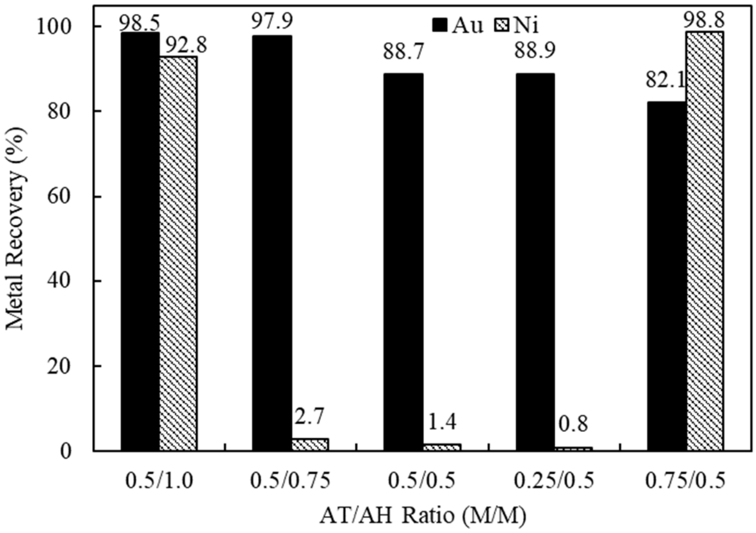

Figure S3. Effect of ratio of ammonium thiosulfate to ammonium hydroxide on recovery of Au and Ni at 240 min (summary of Experiment 9, 11–14).

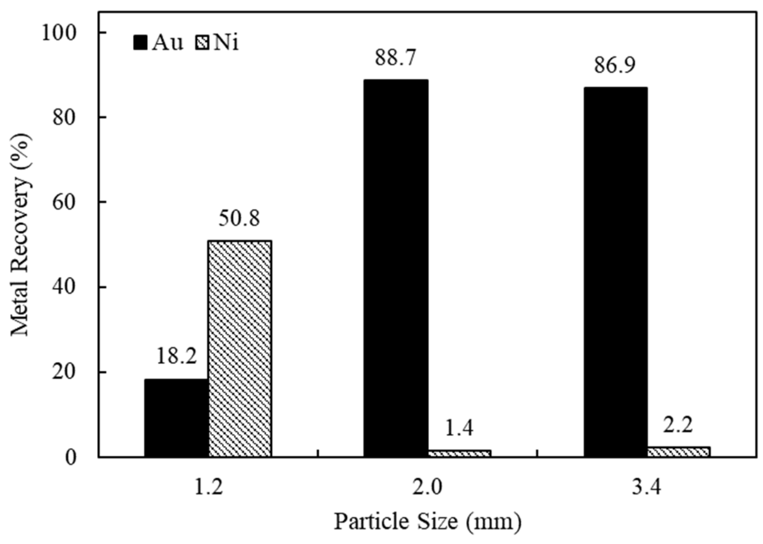

Figure S4. Effect of particle size on the recovery of Au and Ni at 240 min (summary of Experiment 9, 15–16).

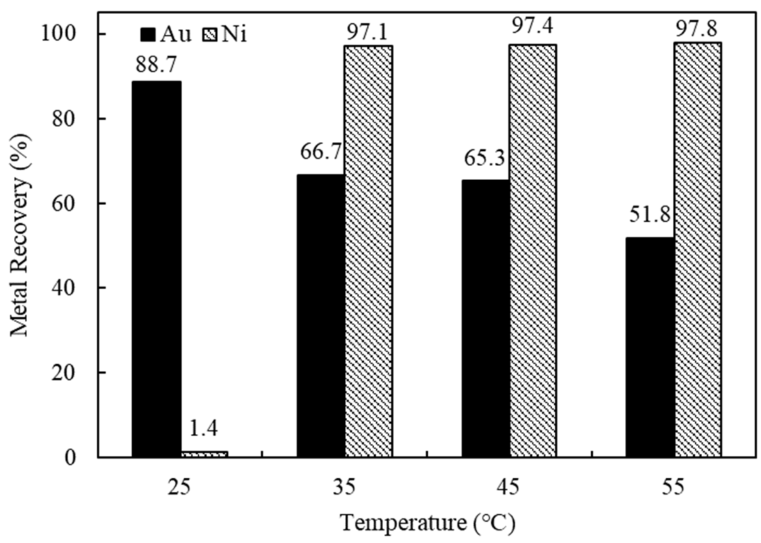

Figure S5. Effect of temperature on the recovery of Au and Ni at 240 min (summary of Experiment 9, 17–19).

Table S1. Au recovery response based on Cu(II) concentration, aeration rate, AT/AH ratio, size and temperature (summarized results from all experiments presented in this study).

Cu(II) (mM)	Aeration (mL/min)	AT/AH ratio (M/M)	Size (mm)	Temp (°C)	Interruption Time (min)	Au Drop Degree (%)
Varying Cu(II) Concentrations (25–75mM) and Aeration Rates (0–120 mL/min)						
25	0	0.5/0.5	2	25	60–240	100%
50	0	0.5/0.5	2	25	60–240	70%
75	0	0.5/0.5	2	25	180–240	35%
25	60	0.5/0.5	2	25	120–240	90%
50	60	0.5/0.5	2	25	>240	0%
75	60	0.5/0.5	2	25	>240	0%
25	120	0.5/0.5	2	25	120–240	80%
50	120	0.5/0.5	2	25	>240	0%
75	120	0.5/0.5	2	25	>240	0%

Varying Ammonium Thiosulfate and Ammonium Hydroxide Concentration Ratio (AT/AH Ratio)						
50	120	0.5/0.5	2	25	>240	0%
50	120	0.5/0.75	2	25	>240	0%
50	120	0.5/1.0	2	25	180–240	<5%
50	120	0.25/0.5	2	25	>240	0%
50	120	0.75/0.5	2	25	15–120	20%
Varying Particle Size (1.2–3.4 mm) and Temperatures (25–55 °C)						
50	120	0.5/0.5	3.4	25	>240	0%
50	120	0.5/0.5	2	25	>240	0%
50	120	0.5/0.5	1.2	25	120–240	60%
50	120	0.5/0.5	2	35	30–120	30%
50	120	0.5/0.5	2	45	<15	0%
50	120	0.5/0.5	2	55	<15	0%

Au Drop Degree	0–10	10–40	40–80	80–100
Interruption Time	>240	120–240	30–120	0–30

Table S2. Table Summary of experimental variables (alternative representation of Table 3).

Increments of Variables	$c[Cu^{2+}]$	Aeration ($21\%O_2$)	$c[S_2O_3^{2-}]/c[NH_3]$ Ratio	Particle Size	Temperature
	mM	mL/min	M/M	mm	°C
3	25, 50, 75	0 (Ar)	0.5/0.5	2.0	25
3	25, 50, 75	60	0.5/0.5	2.0	25
3	25, 50, 75	120	0.5/0.5	2.0	25
3	50	120	0.25/0.5, 0.5/0.5, 0.75/0.5	2.0	25
3	50	120	0.5/0.5, 0.5/0.75, 0.5/1.0	2.0	25
3	50	120	0.5/0.5	3.4, 2.0, 1.2	25
4	50	120	0.5/0.5	2.0	25, 35, 45, 55

Table S3. Coded coefficient table obtained from Minitab factorial regression analysis for aeration rates 60 and 120 mL/min (Experiment 5–10).

Term	Effect	Coef	SE Coef	T-Value	P-Value	VIF
Constant		107.4	10.5	10.20	0.009	
c[Cu2+]	-75.7	-37.8	12.9	-2.93	0.099	1.00
Aeration	-1.6	-0.8	10.5	-0.08	0.946	1.00
c[Cu2+]*Aeration	4.0	2.0	12.9	0.16	0.891	1.00

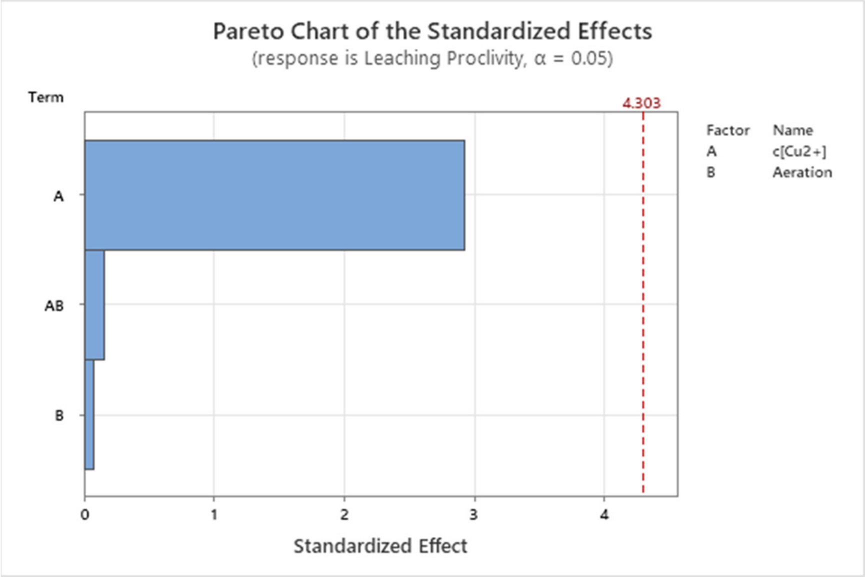

Figure S6. Pareto chart for the copper concentrations and aeration rates 60 and 120 mL/min (Experiment 5–10).

Table S4. Coded coefficient table obtained from Minitab factorial regression analysis for aeration rates 0 and 120 mL/min (Experiment 2–4 and 8–10).

Term	Effect	Coef	SE Coef	T-Value	P-Value	VIF
Constant		142.09	7.75	18.33	0.003	
c[Cu2+]	-138.11	-69.05	9.49	-7.28	0.018	1.00
Aeration	-70.91	-35.45	7.75	-4.57	0.045	1.00
c[Cu2+]*Aeration	66.44	33.22	9.49	3.50	0.073	1.00

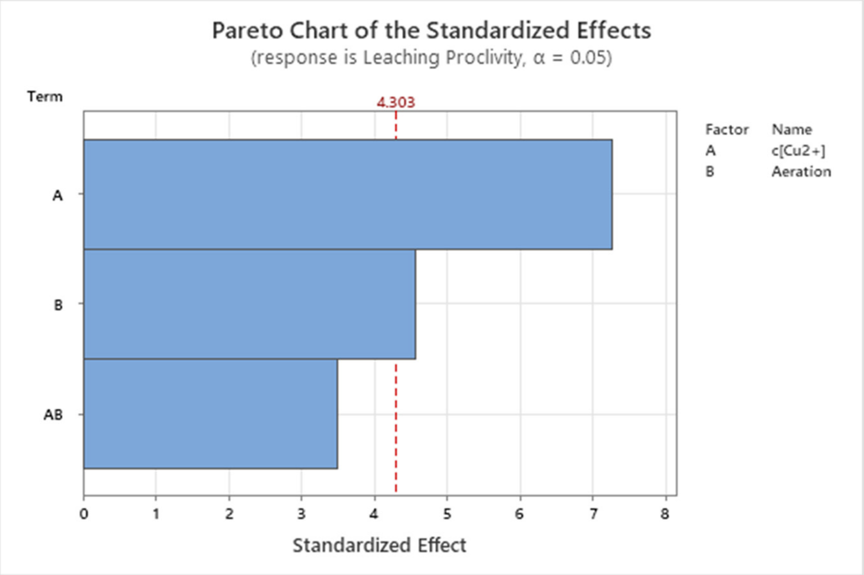

Figure S7. Pareto chart for the copper concentrations and aeration rates 0 and 120 mL/min (Experiment 2–4, 8–10).

Table S5. Analysis of variance table obtained from regression analysis in Minitab for constant thiosulfate concentration when ammonia concentration was varied (Experiment 9, 13, and 14).

Source	DF	SS	MS	F	P
Regression	1	380.698	380.698	27.13	0.121
Error	1	14.034	14.034		
Total	2	394.732			

Table S6. Analysis of variance table for varying concentration of thiosulfate while the ammonia concentration kept constant (Experiment 9, 11, and 12).

Source	DF	SS	MS	F	P
Regression	1	2205.43	2205.43	3.03	0.332
Error	1	727.39	727.39		
Total	2	2932.82			

Table S7. Analysis of variance table for changing concentration of ammonia and thiosulfate (Experiment 9, 11–14).

Source	DF	Adj SS	Adj MS	F-Value	P-Value
Regression	2	1690.7	845.4	4.32	0.188
c[$S_2O_3^{2-}$]	1	966.6	966.6	4.94	0.156
c[NH_3]	1	724.1	724.1	3.70	0.194
Error	2	391.3	195.7		
Total	4	2082.1			

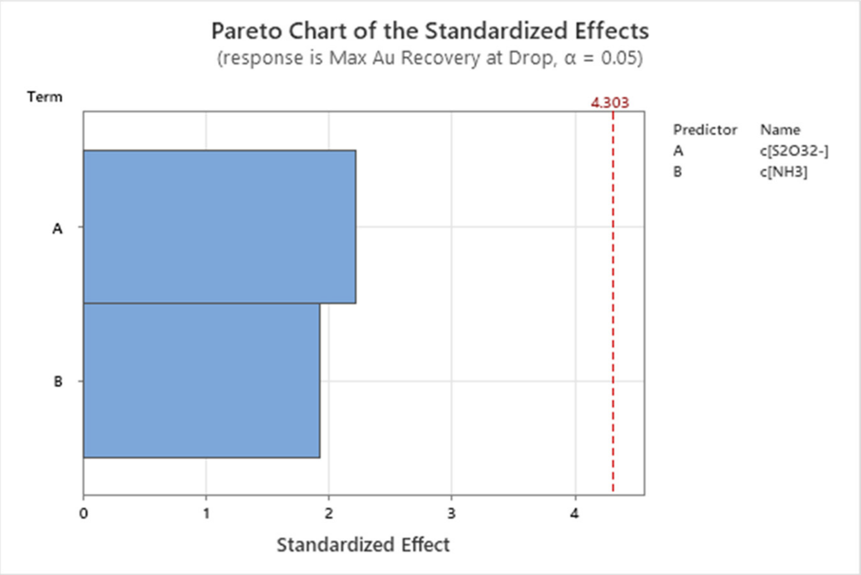

Figure S8. Pareto chart for varying concentrations of ammonia and thiosulfate (Experiment 9, 11–14).

Table S8. Coded coefficient table obtained from Minitab analysis for copper concentration, air gas flow, ammonia thiosulfate concentration, and ammonium hydroxide concentration (Experiment 2–4, 8–16).

Term	Effect	Coef	SE Coef	T-Value	P-Value	VIF
Constant		139.6	48.2	2.90	0.027	
Cu Concentration	-138.1	-69.1	45.2	-1.53	0.177	1.00
Air Gas Flow	-25.4	-12.7	31.1	-0.41	0.697	1.06
AT Conc	390.0	195.0	63.9	3.05	0.022	1.00
AH Conc	-50.4	-25.2	45.2	-0.56	0.597	1.06
Cu Concentration*Air Gas Flow	66.4	33.2	45.2	0.74	0.490	1.00
